# Understanding the Oxidative Properties of Nickel Oxyhydroxide in Alcohol Oxidation Reactions

**DOI:** 10.1021/acscatal.3c01120

**Published:** 2023-06-12

**Authors:** Petrus C. M. Laan, Felix J. de Zwart, Emma M. Wilson, Alessandro Troglia, Olivier C. M. Lugier, Norbert J. Geels, Roland Bliem, Joost N. H. Reek, Bas de Bruin, Gadi Rothenberg, Ning Yan

**Affiliations:** † Van’t Hoff Institute for Molecular Sciences, University of Amsterdam, Science Park 904, 1098 XH Amsterdam, The Netherlands; ‡ Advanced Research Center for Nanolithography (ARCNL), Science Park 106, 1098 XG Amsterdam, The Netherlands; § Key Laboratory of Artificial Micro- and Nano-Structures of Ministry of Education, School of Physics and Technology, Wuhan University, Wuhan 430072, China

**Keywords:** surface reactivity, selective oxidation reactions, single-turnover experiments, kinetics, mechanistic studies, NiOOH

## Abstract

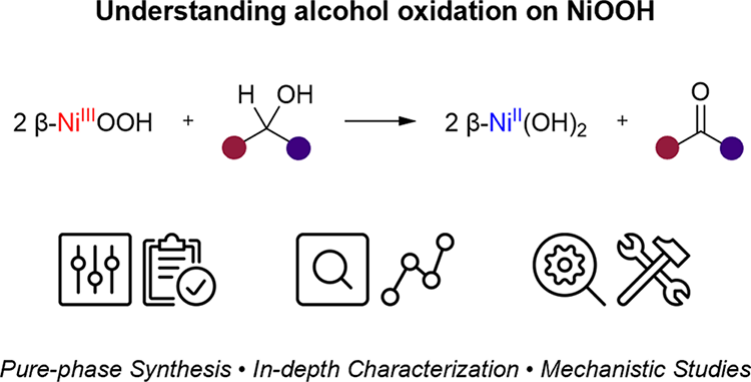

The NiOOH electrode is commonly used in electrochemical alcohol oxidations. Yet understanding the reaction mechanism is far from trivial. In many cases, the difficulty lies in the decoupling of the overlapping influence of chemical and electrochemical factors that not only govern the reaction pathway but also the crystal structure of the *in situ* formed oxyhydroxide. Here, we use a different approach to understand this system: we start with synthesizing pure forms of the two oxyhydroxides, β-NiOOH and γ-NiOOH. Then, using the oxidative dehydrogenation of three typical alcohols as the model reactions, we examine the reactivity and selectivity of each oxyhydroxide. While solvent has a clear effect on the reaction rate of β-NiOOH, the observed selectivity was found to be unaffected and remained over 95% for the dehydrogenation of both primary and secondary alcohols to aldehydes and ketones, respectively. Yet, high concentration of OH^–^ in aqueous solvent promoted the preferential conversion of benzyl alcohol to benzoic acid. Thus, the formation of carboxylic compounds in the electrochemical oxidation without alkaline electrolyte is more likely to follow the direct electrochemical oxidation pathway. Overoxidation of NiOOH from the β- to γ-phase will affect the selectivity but not the reactivity with a sustained >95% conversion. The mechanistic examinations comprising kinetic isotope effects, Hammett analysis, and spin trapping studies reveal that benzyl alcohol is oxidatively dehydrogenated to benzaldehyde *via* two consecutive hydrogen atom transfer steps. This work offers the unique oxidative and catalytic properties of NiOOH in alcohol oxidation reactions, shedding light on the mechanistic understanding of the electrochemical alcohol conversion using NiOOH-based electrodes.

## 1 Introduction

The transition to a sustainable society requires a rethinking of the energy and chemicals sectors.^1,2^ We need to move away from thermochemical processes and fossil carbon sources toward electrosynthesis and using hydrogen as our main energy carrier.^3−8^ This is an enormous challenge, not least because the chemical industry is a mature and conservative one. Yet with change comes opportunity: the direct transfer of electrons to/from substrates can save on reagents and increase overall efficiency. This is especially relevant in the coupling of redox reaction pairs. For example, the oxygen evolution reaction (OER) in a typical water electrolyzer generates low-value oxygen and suffers from sluggish kinetics.^9^ Replacing the OER with an alternative oxidative process promises to coproduce useful chemicals in addition to the hydrogen formed at the cathode.^10−12^ Selective oxidation of alcohols is of great importance in organic synthesis and has been shown as an ideal alternative anodic reaction that produces the corresponding carbonyl and carboxylic compounds.^13^ Unlike the industrial practice which uses costly, and sometimes toxic, stoichiometric oxidants, electrochemical oxidation occurs under mild conditions using green electrons.

NiOOH is perhaps the most popular electrocatalyst employed in these reactions; it often forms *in situvia* the electro-oxidation of a Ni-based precursor such as alloys, hydroxides, phosphides, and sulfides.^14,15^ Despite the rapid progress in NiOOH catalyst development over the past decade, fundamental understanding regarding the alcohol oxidation pathways remains ambiguous. Chemical reactions between the oxyhydroxide and alcohols (also referred to as “indirect oxidation”) to yield carboxylic acids is one widely believed hypothesis, the readily formed Ni(OH)_2_ is then reoxidized electrochemically to sustain continuous alcohol oxidation.^16^ Additionally, there is a potential-dependent reaction mechanism (also referred to as “direct oxidation”) in which an external potential bias must be applied to drive the electrochemical oxidation of substrate. More recently, a hybrid oxidation mechanism, comprising indirect oxidation and direct electrochemical oxidation of alcohols, is proposed in which the alcohol conversion prefers the direct route.^17^ This ambiguity is also reflected by the large selectivity difference in the electrochemical alcohol oxidation: some reports show NiOOH catalyzes the formation of carbonyl compounds with >99% selectivity, while in other work, the conversion to carboxylic compounds prevailed.^18^ In fact, such seemingly contradictory results can be ascribed to the complex electrochemical environment. *e.g.*, the *in situ* formed NiOOH electrode is often ill defined with varied physicochemical properties. For instance, NiOOH generally has β- and γ-phases whose formation and regeneration are dependent on various factors. Although both have layered structures with NiO_2_ sheets and intercalated species, only protons are present in the interlayer space of β-NiOOH while water molecules and metal cations also occupy this space in γ-NiOOH.^19^ Moreover, the Ni oxidation states of these two phases are different. In addition, most of the used KOH electrolyte in the literature is not purified, containing impurities such as Fe which is documented to have a significant influence on the electro-catalytic behaviors of NiOOH. The complex composition of the NiOOH electrode, together with the different electrochemical conditions, makes the elucidation of catalytic behaviors of NiOOH in alcohol oxidation challenging.

In this work, with the aim of qualifying and quantifying the physicochemical factors of NiOOH that govern the alcohol oxidation reaction, we prepared pure β- and γ-NiOOH and examined both their reactivity and selectivity in the oxidation of primary and secondary alcohols. We show that overoxidation of NiOOH from the β- to γ-phase will decrease the overall selectivity of the alcohol oxidation reaction. Moreover, kinetic isotope effect (KIE) studies, Hammett analysis, and spin trapping experiments show that this reaction proceeds *via* two consecutive hydrogen atom transfer steps.

## 2 Results and Discussion

### 2.1 Selective Synthesis and Characterizations of β-/γ-NiOOH

**Figure 1:**
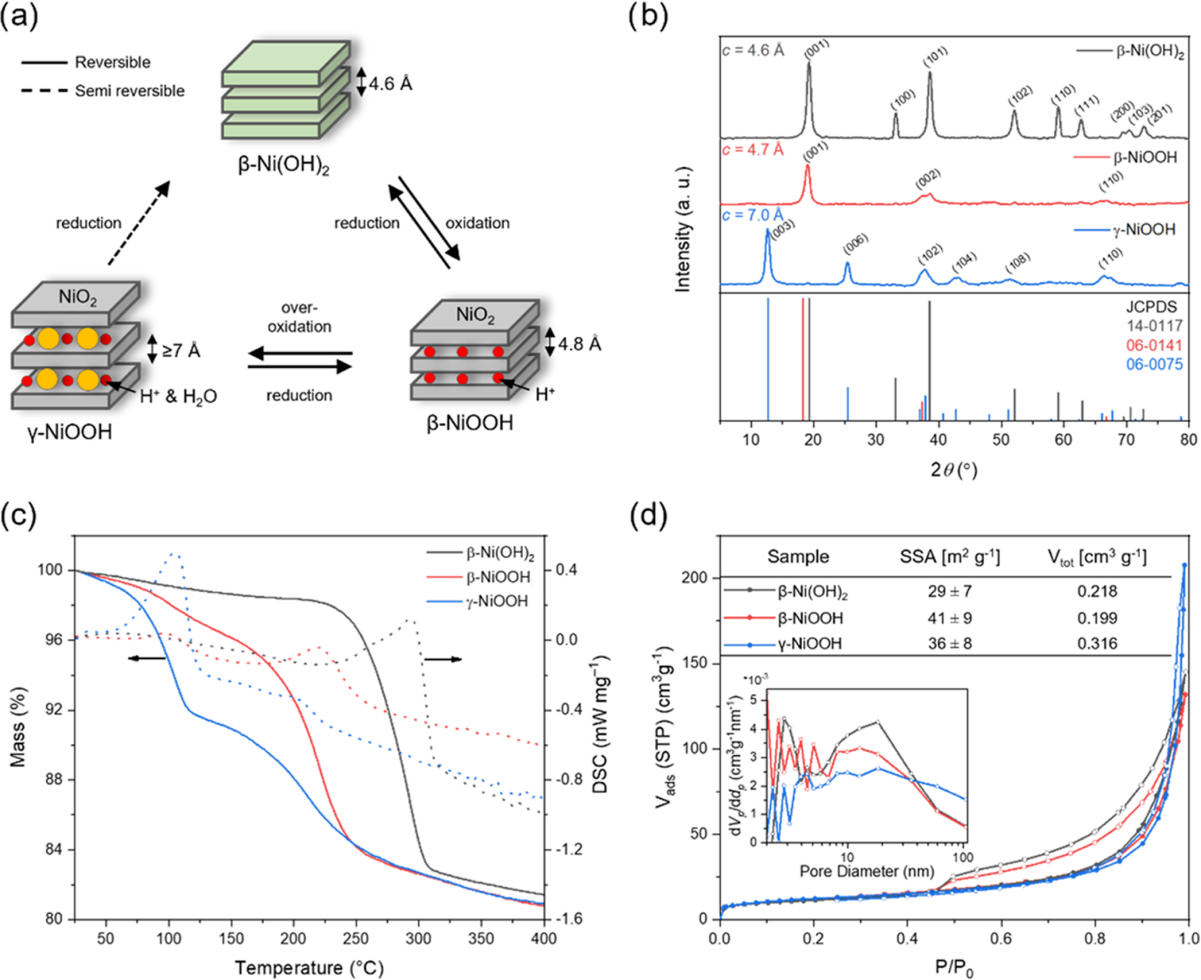
(a) Schematic phase transformations between Ni (oxy)hydroxide phases, (b) powder X-ray diffraction (pXRD) patterns of the obtained materials (top) and the corresponding reference spectra (bottom), (c) thermogravimetric (solid lines) differential scanning calorimetry (dotted lines) (TGA-DSC; 5 K min^–1^, 20 mL min^–1^ Ar) traces, and (d) N_2_ sorption isotherms at 77 K (inset: Barret–Joyner–Halenda (BJH) pore size distribution based on adsorption branches; table: specific surface area (SSA) and total pore volume (*V*_tot_)) of β-Ni(OH)_2_ (black traces), β-NiOOH (red traces) and γ-NiOOH (blue traces).

We developed a simple route of synthesizing pure β-NiOOH and γ-NiOOH (see the schematic structures in Figure 1a). Building on the work of Narayan, we used sodium hypochlorite as the oxidizing agent to form β-NiOOH and γ-NiOOH from β-Ni(OH)_2_. This reagent does not contain transition-metal elements and is easy to wash away after synthesis. In short, using a high concentration of hypochlorite and varying the reaction time between 1 h (β-NiOOH) and 22 h (γ-NiOOH) followed by extensive washing with water are key to the selective synthesis (see Section 4 for details). We then proceeded with a comprehensive characterization. Our objective here was to create a series of standard benchmark values for the different NiOOH phases so that these can be readily compared across studies.

Figure 1b–d shows an overview of the characterization results, and additional characterization figures pertaining to specific items are included in the Supporting Information. The diffraction patterns of all three materials (Figure 1b) are identical to their standard ones, as were the calculated interlayer distances.^20−23^ No other diffraction peaks were observed. The oxyhydroxide peaks are broader than those of the starting material, indicating smaller crystal grains. This was verified by scanning electron microscopy (SEM) imaging (Figure S1). The chemical composition was also confirmed by the energy-dispersive X-ray spectroscopy analysis (EDX, see Figures S2 and S3).

To understand the intercalated species in β- and γ-NiOOH, we then carried out thermogravimetric analysis coupled with differential scanning calorimetry (TGA-DSC). All of the examined materials show a mass loss of *ca.* 19% between 30 and 400 °C (Figure 1c). This reflects the dehydration of β-Ni(OH)_2_ and the respective dehydroxylation of β-NiOOH and γ-NiOOH to NiO (eqs 1 and 2). The formation of NiO at 400 °C was confirmed by pXRD (Figure S4). γ-NiOOH shows more surface water and intercalated water content as the weight loss before 225 °C is much more significant than that of β-NiOOH. This is due to the larger interlayer distance (7.0 Å) compared to β-NiOOH (4.7 Å). This separation weakens the interlayer bonding and enables the hosting of external molecules and cations.^24^ The sharp exothermic peak at *ca.* 225 °C is ascribed to the thermal decomposition of NiOOH, releasing oxygen and water.
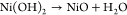
1
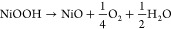
2The higher water content in γ-NiOOH does not come from the higher surface area. In the nitrogen sorption study, β-Ni(OH)_2_ and β-NiOOH show a classical type II isotherm of a nonporous material (Figure 1d).^25^ Furthermore, the H3-type hysteresis suggests a nonlimiting adsorption at high *P*/*P*_0_ values, typical of nonrigid aggregates of plate-like particles. This is in line with the SEM studies, where only plate-like particles are observed (Figures S1–S3). γ-NiOOH has a similar sorption isotherm, but without hysteresis. This indicates the presence of fine macropores, in agreement with the calculated Barrett–Joyner–Halenda (BJH) pore size distribution (see inset in Figure 1d). The calculated specific surface areas (SSAs) based on the Brunauer–Emmett–Teller (BET) theory are all around the 35 m^2^ g^–1^ (Figure 1d).

**Figure 2:**
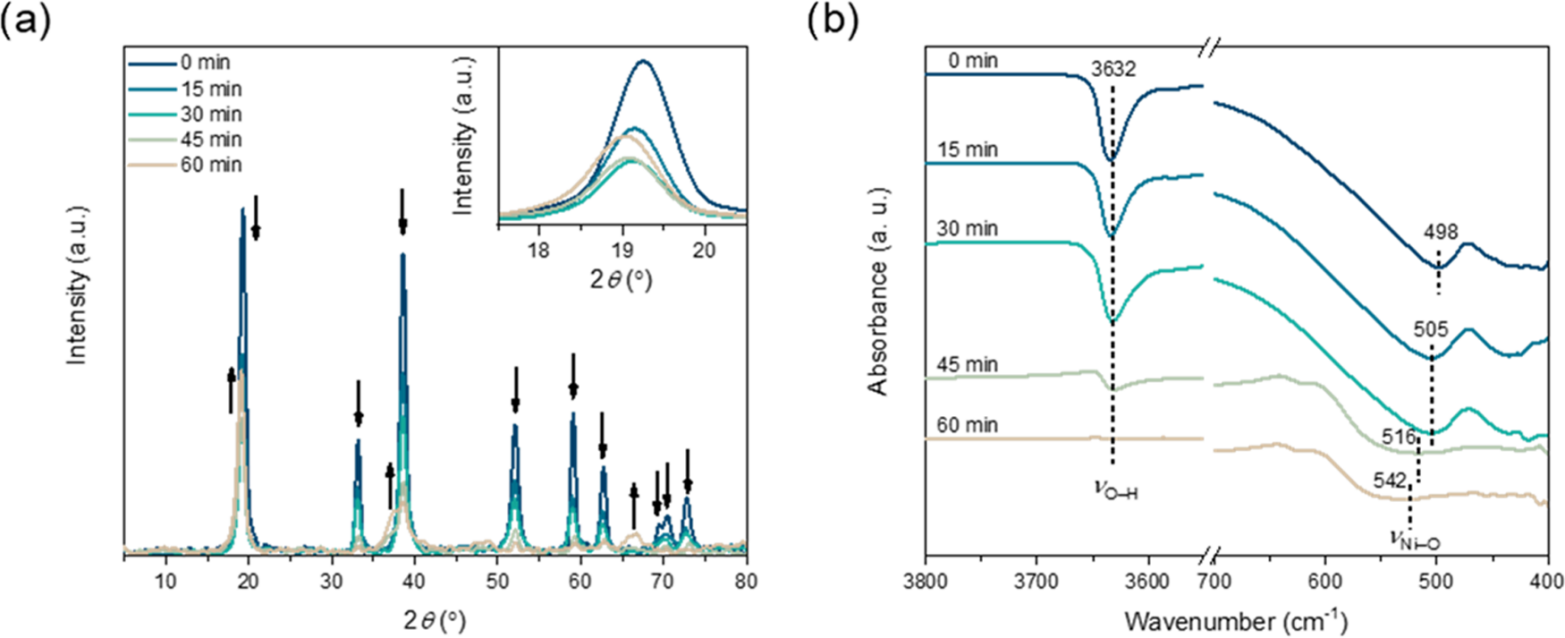
Structural evolution of β-Ni(OH)_2_ into β-NiOOH over time. (a) pXRD patterns and (b) FT-IR spectra.

**Figure 3:**
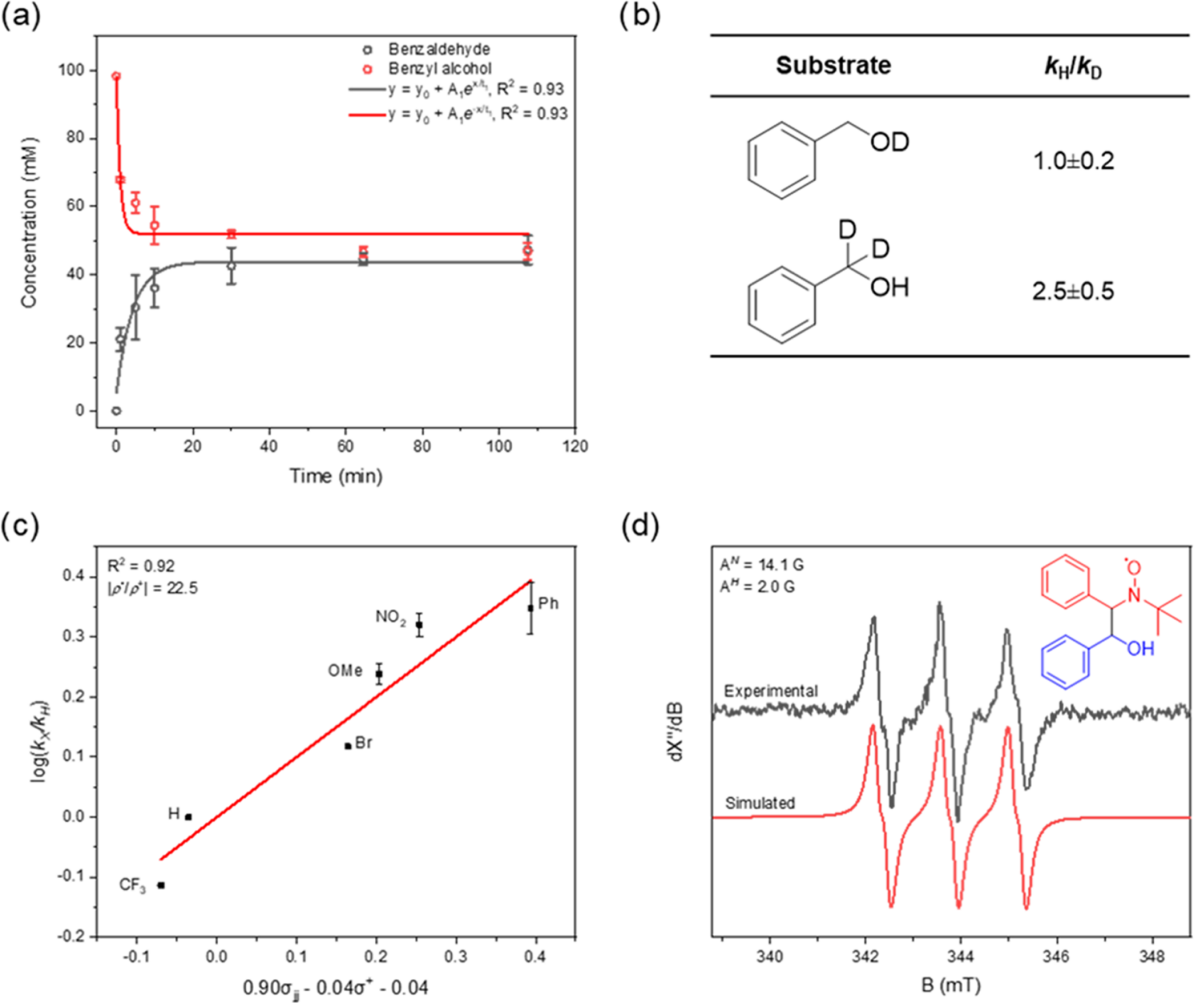
Reactivity and spin trapping studies of the oxidative dehydrogenation of benzyl alcohol. (a) Reaction kinetics at room temperature in toluene. (b) Kinetic isotope effect studies based on initial rate analysis. (c) Hammett plot analysis with varying *para*-substituted benzyl alcohols determined *via* intermolecular competition experiments. (d) Experimental (black) and simulated (red) X-band EPR spectrum at room temperature of PBN*CH(OH)Ph in benzene (A^*N*^ = 14.1 G, A^*H*^ = 2.0 G); the inset shows the chemical structure of the trapped species with PBN in red and benzyl alcohol radical in blue. All reactivity studies were performed at least in duplo. Each data point is the average value, and the error bars are the corresponding standard deviations.

The complete and selective conversion of β-Ni(OH)_2_ to the respective NiOOH phase was also evidenced by the surface analysis using Fourier transform infrared (FT-IR) spectroscopy and X-ray photoelectron spectroscopy (XPS) (Figures S5–S7). β-Ni(OH)_2_ showed both O–H and Ni–O stretches.^26,27^ In the two NiOOH phases, the O–H stretch was absent and the Ni–O stretch was shifted to higher wavenumbers. This result agrees with the high-resolution XPS spectra of the Ni 2p region which reveal a near-quantitative conversion from Ni^2+^ in β-Ni(OH)_2_ to higher-valence Ni in NiOOH.^28^ To avoid the overoxidation of β-NiOOH and to ensure the selective synthesis, we monitored the β-Ni(OH)_2_ → β-NiOOH transformation in greater detail. We did this by sampling at different reaction time during the synthesis of β-NiOOH from β-Ni(OH)_2_. Changes in the bulk and surface structure were studied by pXRD and FT-IR, respectively (Figure 2a,b). We found that β-Ni(OH)_2_ converts gradually to β-NiOOH. The complete disappearance of the hydroxide characteristic XRD peaks occurred after 60 min of oxidation. Note the strongest peak is not a good indicator of the transformation, which overlaps in hydroxide and oxyhydroxide (Figure 3a, inset). The complete surface conversion is more rapid; after 30 min, the FT-IR spectra show only minimal changes (Figure 3b), yet the surface overoxidation to form γ-NiOOH was not prominent (*vide supra*).

### 2.2 Selective Oxidation of Alcohols

Following this characterization, we assessed the oxidative properties of NiOOH by studying the stoichiometric oxidative dehydrogenation of alcohols. Benzyl alcohol was selected as our major model reaction as the number of product is limited, and the mechanistic study is straightforward.^29^ Usually, toluene and water are observed as byproducts, with smaller amounts of benzoic acid, benzyl benzoate, and dibenzyl ether.^30^ The selective formation of the corresponding aldehyde is also of great industrial importance in producing fine chemicals.^13^

The reaction was run in various solvents first to learn how polarity and acidity influence its oxidative behavior (Table 1). We used a 1:2 alcohol/β-NiOOH ratio (see Section 4 for details, results of γ-NiOOH are compared below). Remarkably, no typical byproduct (toluene, benzoic acid, benzyl benzoate, and dibenzyl ether) was found in any of the screened solvents (Table 1, entries 1–7). This shows that the ability to oxidize benzyl alcohol selectively to benzaldehyde is an intrinsic property of β-NiOOH, rather than a solvent effect, in the absence of a secondary catalyst. Notably, the activity differs in different solvents (yield varied from 26 to >95% after 1 h) despite the fact that the NiO–H bond dissociation energy was reported as solvent independent.^31^ As the reaction rates were in the same order of magnitude, a relatively nonpolar reaction is likely (*vide infra*). Apolar solvents provide high yields (>95%), with toluene showing full conversion to the desired aldehyde within 1 h at room temperature (Table 1, entry 7). But there is no clear correlation between polarity and observed yield. This indicates that the reactivity of β-NiOOH in alcohol oxidation reactions is not dictated by solvent polarity alone.

**Table 1:**
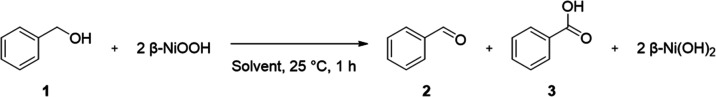
Solvent Screening for the Oxidative Dehydrogenation of Benzyl Alcohol to Benzaldehyde by β-NiOOH^a^

entry	solvent	conversion 1 (%)	yield 2 (%)	selectivity 2 (%)	yield 3 (%)	selectivity 3 (%)
1	D_2_O	26	26	>95	0	0
2	DMF-*d*_7_	26	26	>95	0	0
3	THF-*d*_8_	29	29	>95	0	0
4	acetone-*d*_6_	31	31	>95	0	0
5	CD_3_CN	74	74	>95	0	0
6	benzene-*d*_6_	91	91	>95	0	0
7	toluene-*d*_8_	>95	>95	>95	0	0
8	D_2_O (1M KOH)	56	8	14	48	86

a Conversions and yields are based on ^1^H NMR integration using 1,3,5-trimethoxybenzene (organic solutions) or tetraethylammonium chloride (aqueous solutions) as internal standard.

When high concentrations of OH^–^ ions (1 M KOH) were present in the aqueous solvent, which is the typical alkaline electrolyte used in electrochemical reactions, the conversion increased from 26 to 56% (*cf.*Table 1, entries 1 and 8). Importantly, the reaction pathway changed substantially and the selectivity toward the carboxylate reached 86%. We hypothesize that the promoted hydration of aldehyde under this condition generates gem-diols. Then, the sequential deprotonation and dehydrogenation lead to the formation of gem-diolate and carboxylate, respectively. The base acts as a catalyst, facilitating the formation of carboxylate. Indeed, the reaction *via* this route is less selective, yet proceeds more rapidly. Note that we used nuclear magnetic resonance (NMR) spectroscopy to quantify the content of substrate and products. ±5% accuracy can be easily achieved, so we reported “>95%” when the observed value is literally “100%”. Furthermore, the concurrent transformation from β-NiOOH to β-Ni(OH)_2_ was also found to be selective and quantitative by pXRD (Figure S8) and XPS (Figure S9). Furthermore, SEM-EDX analysis confirmed that the chemical composition, the plate-like morphology of the particles, and their size did not change after reaction (Figure S10).

Using γ-NiOOH as an oxidant instead of β-NiOOH also gave full conversion but a lower benzaldehyde yield (85% vs >95%, see Table 2, entries 1 and 2). No other byproducts were observed in the solvent phase of this reaction. Disproportionation of benzyl alcohol, forming an equimolar mixture of benzaldehyde and toluene, is one of the side reactions known that can explain this observation.^30^ Overoxidation to gaseous CO and CO_2_ is possible in theory, yet unlikely as this complete oxidation is energetically more difficult. The difference of yield is attributable to the reported higher average oxidation state of Ni (3.3–3.67) of this material compared to that in β-NiOOH (2.7–3.0).^32^ The solid residue was analyzed by pXRD (Figure S11). Its pattern could be indexed as β-Ni(OH)_2_ but showed significantly broader peaks compared to the pattern of β-NiOOH after the same reaction (Figure S8). This indicates a more amorphous material which is in line with the poorer reversibility of the β(II)/γ(III) redox couple compared to β(II)/β(III) as documented in battery applications.^33^

**Table 2:**
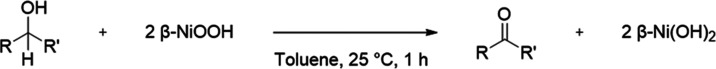
Control Experiments and Broader Applicability of the System in the Alcohol Oxidation^a^

entry	variation from conditions	substrate conversion (%)	yield (%)
1^a^	none	>95	>95
2^a^	γ-NiOOH as oxidant	>95	85
3^a^	β-Ni(OH)_2_ as oxidant	<5	<5
4^a^	no oxidant	<5	<5
5^a^	benzaldehyde as substrate	<5	<5^c^
6^b,f^	cinnamyl alcohol as substrate	79 (1 h), >95 (16 h)	79 (1 h), >95 (16 h)^d^
7^b,f^	1-phenylethanol as substrate	59 (1 h), >95 (16 h)	59 (1h), >95 (16 h)^e^

a Conversions and yields are based on (a) GC-analysis using chlorobenzene in acetonitrile as external standard or (b) ^1^H-NMR integration using 1,3,5-trimethoxybenzene as internal standard. Superscripts refer to (c) benzoic acid-, (d) cinnamaldehyde- and (e) acetophenone yield. (f) Product formation was also confirmed with GC-MS.

Using β-Ni(OH)_2_ as oxidant or no oxidant at all did not show any alcohol conversion (Table 2, entries 3 and 4). These control experiments show that any β-Ni(OH)_2_ that readily forms *viain situ* NiOOH reduction does not cause side reactions. To see whether pure NiOOH would react with the product, benzaldehyde, we ran an experiment using benzaldehyde as the substrate. No conversion was observed (Table 2, entry 5). Based on these results, we can have the following considerations regarding the selective oxidation of benzyl alcohol using NiOOH: (1) Solvent has a significant impact over the reaction rate, but not on the selectivity. (2) Both β- and γ-NiOOH are able to selectively oxidize benzyl alcohol to benzaldehyde with high yield, yet are not sufficiently powerful to enable the aldehyde oxidation to benzoic acid. Thus, in the electrochemical alcohol oxidation using NiOOH catalysts, the selective formation of aldehyde can fully follow the indirect conversion pathway, but the selective formation of carboxylic acid must involve the direct electrochemical mechanism provided that no secondary catalyst (such as base, organometallic complexes, or metal nanoparticles) coexists. (3) γ-NiOOH is the less-desirable catalyst that induced the formation of byproducts. In the electrochemical process, if the *in situ* generated β-NiOOH is not consumed by alcohols in time and is subsequently overoxidized into γ-NiOOH, the aldehyde selectivity is likely to be suppressed. Therefore, efficient mass transfer, *e.g.*, using a flow cell, might be more helpful to counteract this problem.

We then employed β-NiOOH in the selective oxidation of allylic alcohols. The presence of both alkene and primary alcohol functionality has been documented to pose a challenge for the selective conversion.^34^ We selected cinnamyl alcohol oxidation as the model reaction, which often results in the generation of complex products such as 3-phenyl-1-propanol, methylstyrene, and propylbenzene while cinnamaldehyde is the most desirable product. β-NiOOH shows excellent conversion (>95%) and selectivity (>95%) toward cinnamaldehyde (Table 2, entry 6), outperforming many aerobic oxidation processes.^35^ The reaction rate was slower than that of benzyl alcohol, yet complete conversion was achieved after a prolonged reaction time. The high selectivity toward the dehydrogenation of hydroxyl functionality was also validated by using a secondary alcohol. The yield of acetophenone from 1-phenylethanol also topped >95% (Table 2, entries 7). Similar results were also obtained for electron-rich and electron-deficient benzyl alcohols (*vide infra*). Based on these results, we argue that β-NiOOH is highly active and selective toward the activation and dehydrogenation of both primary and secondary alcohol functionalities. The reaction of other functionalities observed during the electrosynthesis using a NiOOH electrode thus must be a potential-dependent process.

### 2.3 Mechanistic Insight of the Selective Oxidation

To understand the oxidative and catalytic behavior of β-NiOOH better in selective oxidation, we ran mechanistic studies using benzyl alcohol conversion as an example. We started by measuring the reaction kinetics, thus building a framework for designing experiments to test a proposed mechanism. These experiments were run using a 1:1 alcohol/β-NiOOH ratio (Figure 3a). The results show that the reaction is first order in substrate and reaches maximum conversion after 30 min at room temperature. The observation that alcohol conversion and aldehyde yield both approach 50% demonstrates that the stoichiometric ratio in which benzyl alcohol and β-NiOOH react is indeed 1:2.

To gain further insight into the reaction intermediates, we performed kinetic isotope effects (KIEs) studies, Hammett analysis, and spin trapping experiments (Figure 3b–d). We used the initial rates method to determine the KIEs using benzyl alcohol-*d*_1_ (Ph-CH_2_-OD) and benzyl alcohol-*d*_2_ (Ph-CD_2_-OH). The respective KIEs were as *k*_OH_/*k*_OD_ = 1.0 ± 0.2 and *k*_CH_2__/*k*_CD_2__ = 2.5 ± 0.5 (Figure 3b). We conclude that α-hydrogen atom abstraction from benzyl alcohol is the rate-limiting step.^36^ The latter KIE value also indicates a hydrogen atom transfer step.^37,38^

To obtain information about the electronic structure of this rate-liming transition state, we ran a Hammett analysis using intermolecular competition experiments between benzyl alcohol and *para*-substituted (OMe, Ph, H, Br, CF_3_, and NO_2_) benzyl alcohols (Figure 3c, see Section 4 for details). We used a substoichiometric amount (0.2 equiv) of β-NiOOH to ensure benzyl alcohol conversion remains low (<20%) so that the ratio between the nonsubstituted and substituted benzaldehyde reflects their relative reaction rate, *i.e.*, *k*_H_/*k*_X_. Besides the classical electronic Hammett constants (σ^+^ and σ^–^) defined by Taft et al.,^39^ we included also the radical spin-delocalization substituent constants (σ*_jj_*•) defined by Jiang^40^ to account for possible radical-type contributions to the transition state. The best fit was found for {ρ^•^σ*_jj_*^•^ + ρ^+^σ^+^ + *C*} by multiple linear regression (*R*^2^ = 0.92 for six observations, ρ^•^ = 0.90, ρ^+^ = −0.04 and *C* = −0.04). The large Hammett |ρ^•^/ρ^+^| ratio (22.5) shows that radical stabilization effects dictate the reactivity of benzyl alcohol rather than classical electronic effects. Therefore, we can say that the rate-limiting transition state is controlled by the delocalization of spin density over the benzyl alcohol fragment.^40^

To get more information about the radical intermediate, we finally ran spin trapping studies with *N*-*tert*-butyl-α-phenylnitrone (PBN). A solution of benzyl alcohol was briefly stirred with β-NiOOH after which the reaction mixture was quickly filtered onto a solution of PBN. X-band electron paramagnetic resonance (EPR) analysis of the PBN-trapped radical intermediate showed one single paramagnetic component (Figure 3d). This component could be assigned to the carbon-centered benzyl alcohol radical based on the hyperfine splitting interactions (literature *A*^*N*^ = 14.2 G, *A*^*H*^ = 2.1 G, found *A*^*N*^ = 14.1 G, *A*^*H*^ = 2.0 G).^41^ This further supports our hypothesis that the first step is indeed α-hydrogen abstraction from benzyl alcohol *via* hydrogen atom transfer.

**Scheme 1:**
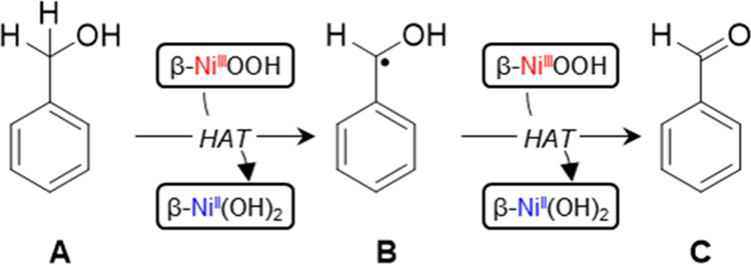
Proposed Mechanism for the Oxidative Dehydrogenation of Benzyl Alcohol (**A**) *via* the Carbon-Centered Benzyl Alcohol Radical (**B**) to Benzaldehyde (**C**) by β-NiOOH *via* Two Hydrogen Atom Transfer (HAT) Steps with Concurrent Conversion of β-NiOOH to β-Ni(OH)_2_

Based on our mechanistic work and literature reports, we propose the mechanism as depicted in Scheme 1. The dehydrogenation of benzyl alcohol starts with hydrogen transfer from the substrate to β-NiOOH to form the carbon-centered benzyl alcohol radical **B** and the first equivalent of β-Ni(OH)_2_. The second hydrogen transfer occurs in a similar fashion to generate benzaldehyde **C** and the second equivalent of β-Ni(OH)_2_. In short, benzyl alcohol is oxidatively dehydrogenated to benzaldehyde *via* two consecutive hydrogen atom transfer steps.

## 3 Conclusions

*Via* synthesizing pure compounds of both NiOOH phases, we show that β-NiOOH is highly reactive (>95% conversion) and selective (>95% selectivity) for the dehydrogenation of both primary and secondary alcohols toward the formation of aldehydes and ketones, respectively; γ-NiOOH, however, can induce the formation of byproducts in benzyl alcohol oxidation. Thus, overoxidation of NiOOH electrode from β to γ phase will affect the overall selectivity. Moreover, the solvent has a clear effect on the reaction rate, yet shows little influence over the reaction selectivity. In particular, a high concentration of OH^–^ in the aqueous solvent promoted the preferential conversion of benzyl alcohol to benzoic acid. The mechanistic study reveals that benzyl alcohol is oxidatively dehydrogenated to benzaldehyde *via* two consecutive hydrogen atom transfer steps. Based on these insights, we maintain that the formation of carbonyl compounds on pure NiOOH electrodes could completely follow the indirect pathway; the formation of carboxylic compounds is more likely to follow the direct electrochemical oxidation pathway without secondary catalysts (such as alkaline electrolyte, organometallic complexes, or metal nanoparticles). Apparently, more work must be done in the future to decouple the chemical and electrochemical factors that affect the roles of NiOOH, particularly in the scenario where the electrochemical regeneration process of metal oxyhydroxide can also greatly affect the activity of the electrode. This work shows the unique oxidative properties of NiOOH in alcohol oxidation reactions and provides a mechanistic understanding of the electrochemical alcohol conversion using NiOOH electrodes.

## 4 Experimental Section

### 4.1 General Considerations

All chemicals were purchased from commercial sources and used as received unless stated otherwise. Specifically, sodium hypochlorite (5 L, 14% Cl_2_ in aqueous solution, 2 M) was obtained from VWR chemicals, β-Ni(OH)_2_ (250 g, 60.0–70.0% Ni basis) was obtained from Sigma-Aldrich, and *N*-*tert*-butyl-α-phenylnitrone (PBN) was obtained from Alfa Aesar GmbH & Co KG and stored at −30 °C in a N_2_-filled glovebox (MBraun Unilab). Benzyl alcohol-*d*_1_ (Ph-CH_2_-OD)^42^ and benzyl alcohol-*d*_2_ (Ph-CD_2_-OH)^43^ were prepared according to literature procedures.

### 4.2 Powder X-ray Diffraction

Powder X-ray diffraction (pXRD) patterns were obtained with a MiniFlex II diffractometer using Ni-filtered Cu Kα radiation (λ = 1.541874 Å) at 30 kV and 15 mA. For each measurement, the sample was ground and loaded on a monocrystalline silicon sample holder with an 8 mm wide and 0.2 mm deep cavity. The powdered sample was pressed firmly in the cavity to make a uniform flat sample area. Residual sample outside the sample cavity was removed to minimize background scattering. Diffraction patterns were collected in the 2θ range of 5 and 80° using a rotation speed of 2° min^–1^, a step size of 0.05°, and 1 s dwell time.

### 4.3 Scanning Electron Microscopy

Scanning electron microscopy (SEM) and energy-dispersive X-ray (EDX) mapping were performed on an FEI Verios 460 (using 5 kV electrons) equipped with an Oxford Xmax 80 mm^2^ silicon drift detector. Samples were dispersed in ethanol (±0.01 mg in 1 mL) by sonication for 1 h before drop-casting on silicon wafers.

### 4.4 X-ray Photoelectron Spectroscopy

X-ray photoelectron spectroscopy (XPS) data were acquired using a Scienta Omicron HiPP-3 analyzer and a monochromatic Al Kα source operating at 20 mA emission current. The base pressure was about 2 × 10^–9^ mbar, and the operating pressure was about 5 × 10^–9^ mbar. Survey and high-resolution spectra were acquired at pass energies of 500 and 100 eV, respectively. Charge accumulation at the surface caused substantial sample-dependent shifts of the electron kinetic energy, which require an energy-dependent correction of the peak position. The Ni 2p_3/2_ spectra were therefore aligned to the literature values of the majority species observed by pXRD.^28^ Compared to calibration *via* the C 1s peak of adventitious carbon, this approach deviates by up to 0.7 eV. The XPS spectra were deconvoluted using the software KolXPD, employing a Shirley background and Voigt functions.

### 4.5 Thermogravimetric Analysis–Differential Scanning Calorimetry

Thermogravimetric analysis–differential scanning calorimetry (TGA-DSC) was carried out using a NETZSCH Jupiter STA 449F3 instrument. The measurements were made under a flow of argon (20 mL min^–1^) in the temperature range of 30–400 °C, using a scan rate of 5 °C min^–1^. Approximately 10 mg of each sample was analyzed to minimize heat transfer problems through the sample.

### 4.6 N_2_ Adsorption–Desorption

N_2_ adsorption–desorption isotherms were measured using a Thermo Scientific Surfer instrument at 77 K, using vacuum-dried samples. Around 100 mg of each sample was dried at 100 °C for 16 h on a Belprep-vacIII prior to analysis. Isotherms were analyzed by the Thermo Fischer Advanced Data Processing 6.0 software, using the BET2 model for specific surface area.^44^ The specific surface area was determined based on the adsorption branch and the BET analysis was performed according to the Rouquerol consistency criteria (Figures S12–S14).^45,46^ The pore size distributions were estimated according to the desorption branch of the isotherm using the Barrett–Joyner–Halenda (BJH) model.^47^

### 4.7 Electron Paramagnetic Resonance

Electron paramagnetic resonance (EPR) spectra of the samples were measured in EPR quartz tubes on a Bruker EMX-plus CW X-band spectrometer at room temperature (298 K). The spectra were obtained on freshly prepared solutions and simulated using EasySpin *via* the cwEPR GUI.^48^

### 4.8 Fourier Transform Infrared

Fourier transform infrared (FT-IR) spectra were collected using a Thermo Scientific Nicolet iS50 FT-IR spectrometer between 4000 and 400 cm^–1^ with a resolution of 4 cm^–1^ after 32 scans per spectrum using a Specac Quest high-throughput Attenuated Total Reflection accessory. Samples were analyzed directly without any dilution.

### 4.9 Gas Chromatography

Gas chromatography (GC) analysis was carried out using a PerkinElmer Clarus 580 Gas chromatograph equipped with an Agilent Technologies, Inc. HP-5 column (30 m × 0.32 mm i.d. × 0.25 μm film thickness) and a flame ionization detector. The temperature program was as follows: initial temperature = 100 °C, 1 min; final temperature = 340 °C, 5 min; heating rate = 30 °C min^–1^. The temperature of the injector was 280 °C, and the temperature of the detector was 280 °C. Before each run, the injection needle was flushed three times with acetonitrile to prevent any cross-contamination. These GC operating parameters allowed for the retention peaks of the acetonitrile, toluene, chlorobenzene, benzaldehyde, benzyl alcohol, and benzoic acid to be baseline separated with retention times of 2.43, 2.84, 3.09, 3.60, 3.99, and 5.19 min, respectively. Chlorobenzene was added as a stock solution in acetonitrile to aliquots and used as an external standard for quantification.

### 4.10 Nuclear Magnetic Resonance

Nuclear magnetic resonance (NMR) spectra were recorded on a Bruker AVANCE III HD 300 MHz spectrometer or on a Bruker AVANCE III HD 500 at room temperature and referenced to the solvent residual signal (7.09, 7.01, 6.97, and 2.08 for toluene) and converted to the TMS scale. For quantitative ^1^H NMR, 1,3,5-trimethoxybenzene was used as internal standard. Data was processed and visualized using MestReNova 10.0.2.

### 4.11 Gas Chromatography–Mass Spectrometry

Gas chromatography–mass spectrometry (GC-MS) analysis was performed on a Shimadzu GC-MS-CP2010 SE plus equipped with a Shimadzu SH-Rtx-5 Amine column (30 m × 0.25 mm i.d. × 0.25 μm film thickness) and a flame ionization detector. The temperature program was as follows: initial temperature = 50 °C, 4 min; ramp to 150 °C with 10 °C min^–1^; ramp to 300 °C with 50 °C min^–1^; final temperature = 300 °C, 3 min. Temperature of injector = 250 °C, temperature of detector = 330 °C. Before each run, the injection needle was flushed two times with methanol to prevent any cross-contamination. Mass spectrometry was obtained by electronic impact (EI) with the ion source at 260 °C and the detector voltage set at 70 eV. Spectra were obtained in the positive mode after a solvent cut time of 2.9 min over a range of 35–600 *m*/*z*.

### 4.12 Procedure for β-NiOOH and γ-NiOOH Preparation

Nickel oxyhydroxide powder was prepared using a modified literature procedure.^49^*Caution*: chlorine gas will evolve during this synthesis, always ensure appropriate ventilation (eq 3)
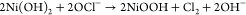
3Green β-Ni(OH)_2_ powder (1.00 g, 10.78 mmol, 1 equiv) was added to a 2.5 L three-neck round-bottom flask. Sodium hypochlorite aqueous solution (1 L, 1.97 mol, 185 equiv) was added, and the suspension quickly turned black. Thereafter, the reaction mixture was left stirring for 1 h at room temperature, after which the reaction was filtered with a Nylon filter under vacuum for 1 h. For γ-NiOOH synthesis, the solution was filtered with a glass frit for 22 h. Both resulting black solids were washed with deionized water until the washings were pH-neutral (±800 mL). The black solid was dried in a vacuum oven for 2 h (50 °C, 5 mbar) and used without further purification.

### 4.13 General Procedure for Alcohol Dehydrogenation Reactions

A 4 mL single-use vial was charged with stock solutions of an alcohol (100 mM, 0.50 mL, 0.05 mmol, 1.0 equiv) and 1,3,5-trimethoxybenzene (20 mM, 0.50 mL, 0.01 mmol) in DCM. The solvent was allowed to evaporate overnight at room temperature under atmospheric pressure. Afterward, toluene-*d*_8_ (1 mL) was added to the vial which was left to stir at room temperature for 1 h yielding all colorless solutions. A different 4 mL single-use vial was charged with a stirring bar (3 mm × 10 mm) and β-NiOOH (9.63 mg, 0.105 mmol, 2.1 equiv). To this vial, the solution of alcohols and 1,3,5-trimethoxybenzene was added. Full dispersion of β-NiOOH was achieved after sonication (1 min) after which the reaction mixture was allowed to proceed at room temperature for 1 or 16 h(s). The reaction mixtures were centrifuged (4000 rpm, 4 min) before an aliquot (0.6 mL) was taken for NMR analysis and afterward used for GC-MS analysis.

### 4.14 Solvent Screening

A 4 mL single-use vial was charged with stock solutions of benzyl alcohol (100 mM, 0.50 mL, 0.05 mmol, 1.0 equiv) and 1,3,5-trimethoxybenzene (20 mM, 0.50 mL, 0.01 mmol) in DCM for organic solvents and with tetraethylammonium chloride (10 mM, 0.50 mL, 0.005 mmol) for aqueous solvents. The solvent was allowed to evaporate overnight at room temperature under atmospheric pressure. Afterward, the solvent of interest (1 mL of D_2_O, DMF-*d*_7_, acetone-*d*_6_, CD_3_CN, THF-*d*_8_, benzene-*d*_6_, or toluene-*d*_8_) was added to the vial which was left to stir at room temperature for 1 h yielding all colorless solutions. A different 4 mL single-use vial was charged with a stirring bar (3 mm × 10 mm) and β-NiOOH (9.63 mg, 0.105 mmol, 2.1 equiv). To this vial, the solution of benzyl alcohol and internal standard was added. Full dispersion of β-NiOOH was achieved after sonication (1 min) after which the reaction mixture was allowed to proceed at room temperature for 1 h. The reaction mixtures were centrifuged (4000 rpm, 4 min) before an aliquot (0.6 mL) was taken for NMR analysis.

### 4.15 Kinetic Studies

A 4 mL single-use vial was charged with a stock solution of benzyl alcohol (100 mM, 1.0 mL, 0.1 mmol, 1.0 equiv) in DCM. The solvent was allowed to evaporate overnight at room temperature under atmospheric pressure. Afterward, toluene (2 mL) was added to the vial which was left to stir at room temperature for 1 h yielding a colorless solution. A different 4 mL single-use vial was charged with a stirring bar (3 mm × 10 mm) and β-NiOOH (19.26 mg, 0.21 mmol, 2.1 equiv). To this vial, the solution of benzyl alcohol was added. Full dispersion of β-NiOOH was achieved after sonication (1 min) after which the reaction mixture was allowed to proceed at room temperature for 2 h. Aliquots (0.1 mL) were taken after 1, 5, 10, 30, 70, and 120 min and combined with a stock solution of chlorobenzene in acetonitrile (9.83 mM, 0.9 mL, 0.0088 mmol) for GC-analysis. Experiment was performed in triplo.

### 4.16 Kinetic Isotope (KIE) Studies

A 4 mL single-use vial was charged with a stock solution of benzyl alcohol, benzyl alcohol-*d*_1_, or benzyl alcohol-*d*_2_ (100 mM, 0.50 mL, 0.05 mmol, 1.0 equiv) in DCM. The solvent was allowed to evaporate overnight at room temperature under atmospheric pressure. Afterward, toluene (1 mL) was added to the vial which was left to stir at room temperature for 1 h yielding a colorless solution. A different 4 mL single-use vial was charged with a stirring bar (3 mm × 10 mm) and β-NiOOH (9.63 mg, 0.105 mmol, 2.1 equiv). To this vial, the solution of benzyl alcohol was added. Full dispersion of β-NiOOH was achieved after sonication (1 min). Aliquots (0.1 mL) were taken immediately to ensure that the conversion of benzyl alcohols was well below 50% and combined with a stock solution of chlorobenzene in acetonitrile (9.83 mM, 0.9 mL, 0.0088 mmol) for GC-analysis. Experiments were performed in duplo.

### 4.17 Hammett Parameter Studies

A 4 mL single-use vial was charged with stock solutions of nonsubstituted benzyl alcohol (100 mM, 0.25 mL, 0.025 mmol, 0.5 equiv), *para*-substituted benzyl alcohol (X = OMe, Ph, H, Br, CF_3_, and NO_2_) (50 mM, 0.50 mL, 0.025 mmol, 0.5 equiv), and 1,3,5-trimethoxybenzene (20 mM, 0.50 mL, 0.01 mmol) in DCM. The solvent was allowed to evaporate overnight at room temperature under atmospheric pressure. Afterward, toluene-*d*_8_ (1 mL) was added to the vial which was left to stir at room temperature for 1 h yielding a colorless solution. A different 4 mL single-use vial was charged with a stirring bar (3 mm × 10 mm) and substoichiometric amounts of β-NiOOH (1.93 mg, 0.021 mmol, 0.2 equiv). To this vial, the solution of benzyl alcohols and 1,3,5-trimethoxybenzene was added. Full dispersion of β-NiOOH was achieved after sonication (1 min) after which the reaction mixture was allowed to proceed at room temperature for 4 h. The reaction mixtures were centrifuged (4000 rpm, 4 min) before an aliquot (0.6 mL) was taken for NMR analysis. Experiments were all performed in duplo. The average ratio of *para*-substituted benzyl alcohol and benzyl alcohol was determined by quantitative ^1^H-NMR spectroscopy using 1,3,5-trimethoxybenzene as an internal standard. This ratio directly equates to the ratio of *k*_H_/*k*_X_ as the substoichiometric amount of β-NiOOH ensures benzyl alcohol conversion <20%. The log (*k*_H_/*k*_X_) values were plotted versus ρ^•^σ*_jj_*^•^, ρ^+^σ^+^ or ρ^–^σ^–^ or combinations hereof and fitted by multiple linear regression. σ_jj_^•^ values were obtained from Jiang,^40^ and σ^+^σ^–^ values were obtained from Taft and co-workers.^39^

### 4.18 Spin Trapping Studies

A flame-dried 5 mL Schlenk flask was charged with a stirring bar (3 mm × 10 mm), β-NiOOH (9.63 mg, 0.105 mmol, 2.1 equiv), benzyl alcohol (5.2 μL, 0.05 mmol, 1.0 equiv), and predried and degassed benzene (1 mL) under Ar. Full dispersion of β-NiOOH was achieved after sonication (1 min). After 10 min, an aliquot (0.5 mL) was transferred over a syringe filter to a different flame-dried 5 mL Schlenk flask, which was charged with a stirring bar (3 mm × 10 mm) and a solution of PBN (13.3 mg, 0.075 mmol, 3.0 equiv) in benzene (0.3 mL) under Ar. The resulting colorless solution was taken for EPR analysis.

## Supporting Information Available

SEM images, EDX elemental mappings, additional pXRD spectra, additional FT-IR spectra, XPS spectra (both survey and Ni 2p_3/2_) and fittings, and Rouquerol-based porosity analysis (PDF)
