# Supplementary material for: Understanding the Oxidative Properties of Nickel Oxyhydroxide in Alcohol Oxidation Reactions
Source: ACS Catal. 2023 Jun 12;13(13):8467–76. doi: 10.1021/acscatal.3c01120 (PMC10334462; doi:10.1021/acscatal.3c01120)
Supplement: Supplementary file 1 — cs3c01120_si_001.pdf [file cs3c01120_si_001.pdf]

Understanding the oxidative properties of nickel oxyhydroxide in alcohol oxidation reactions

Petrus C. M. Laan [a], Felix J. de Zwart [a], Emma M. Wilson [a], Alessandro Troglia [b], Olivier C. M. Lugier [a], Norbert J. Geels [a], Roland Bliem [b], Joost N. H. Reek [a], Bas de Bruin [a], Gadi Rothenberg [a,*] and Ning Yan [a,c*]

[a] Van ’t Hoff Institute for Molecular Sciences, University of Amsterdam, Science Park 904, 1098 XH Amsterdam, The Netherlands.

[b] Advanced Research Center for Nanolithography (ARCNL), Science Park 106, 1098XG Amsterdam, The Netherlands.

[c] Key Laboratory of Artificial Micro- and Nano-Structures of Ministry of Education, School of Physics and Technology, Wuhan University, Wuhan, 430072, China.

E-mail: g.rothenberg@uva.nl (GR), ning.yan@whu.edu.cn (NY)

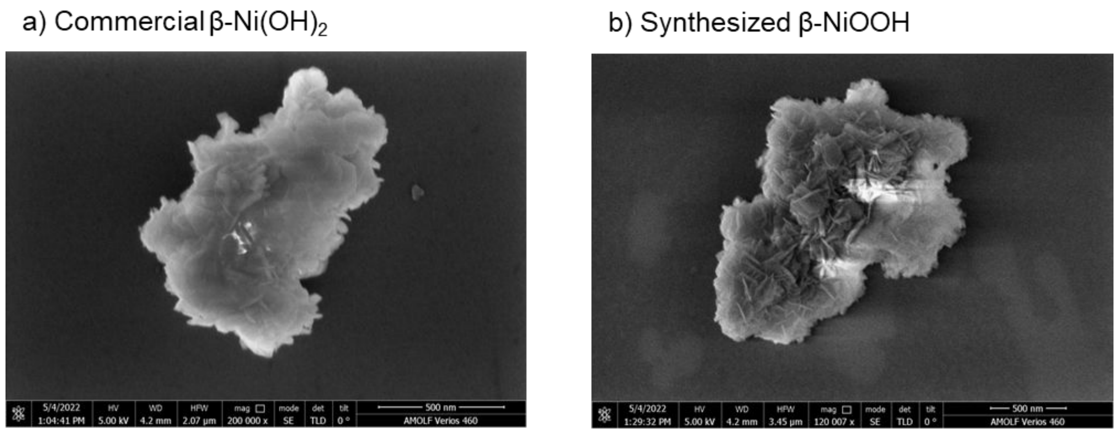

Figure S1. SEM images of a) commercial β-$Ni(OH)_2$ and b) synthesized β-NiOOH. See Figures S2 and S3 for the corresponding EDX mapping.

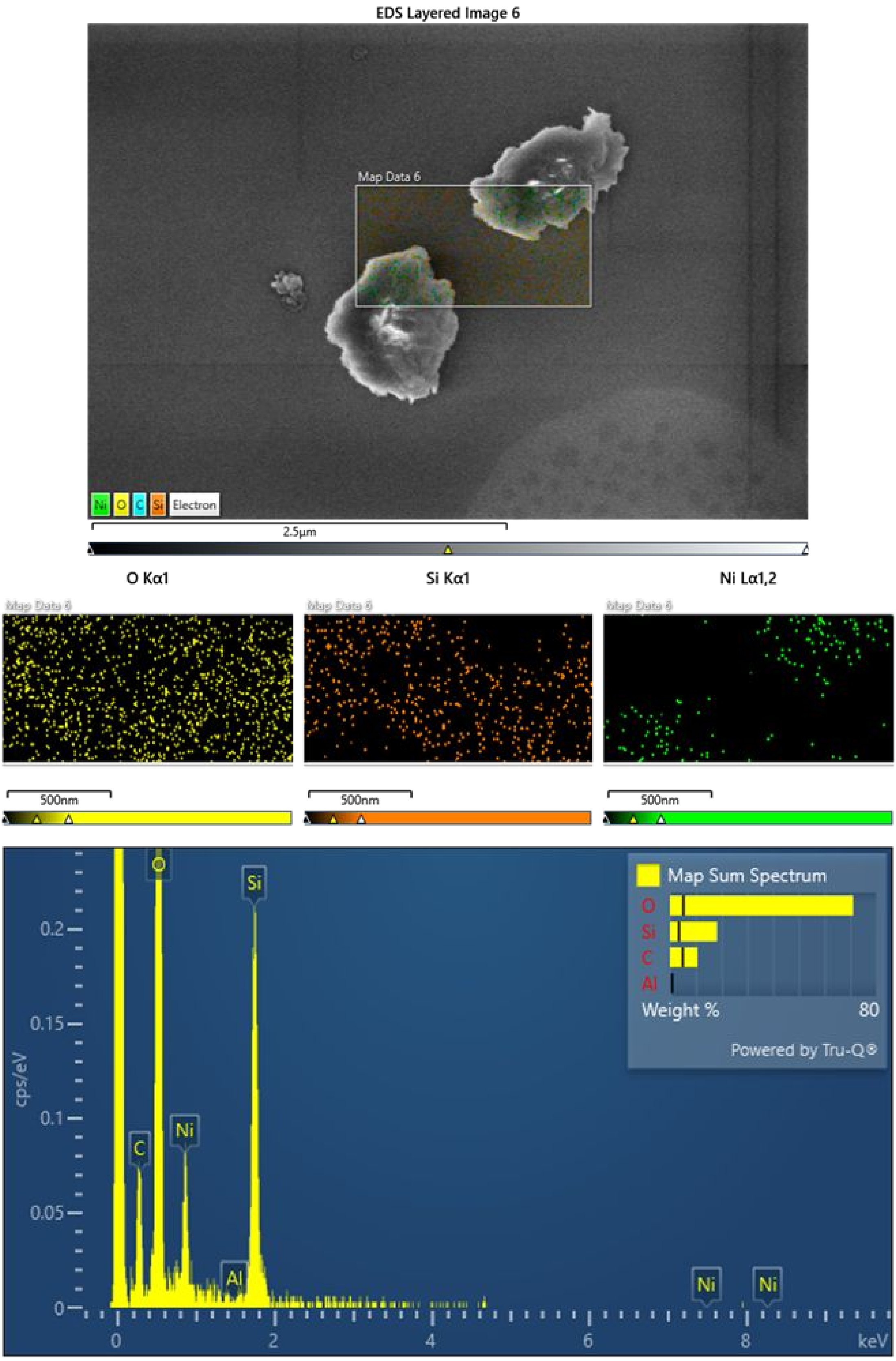

Figure S2. SEM image (top) and corresponding EDX mapping (bottom) of commercial β-$Ni(OH)_2$.

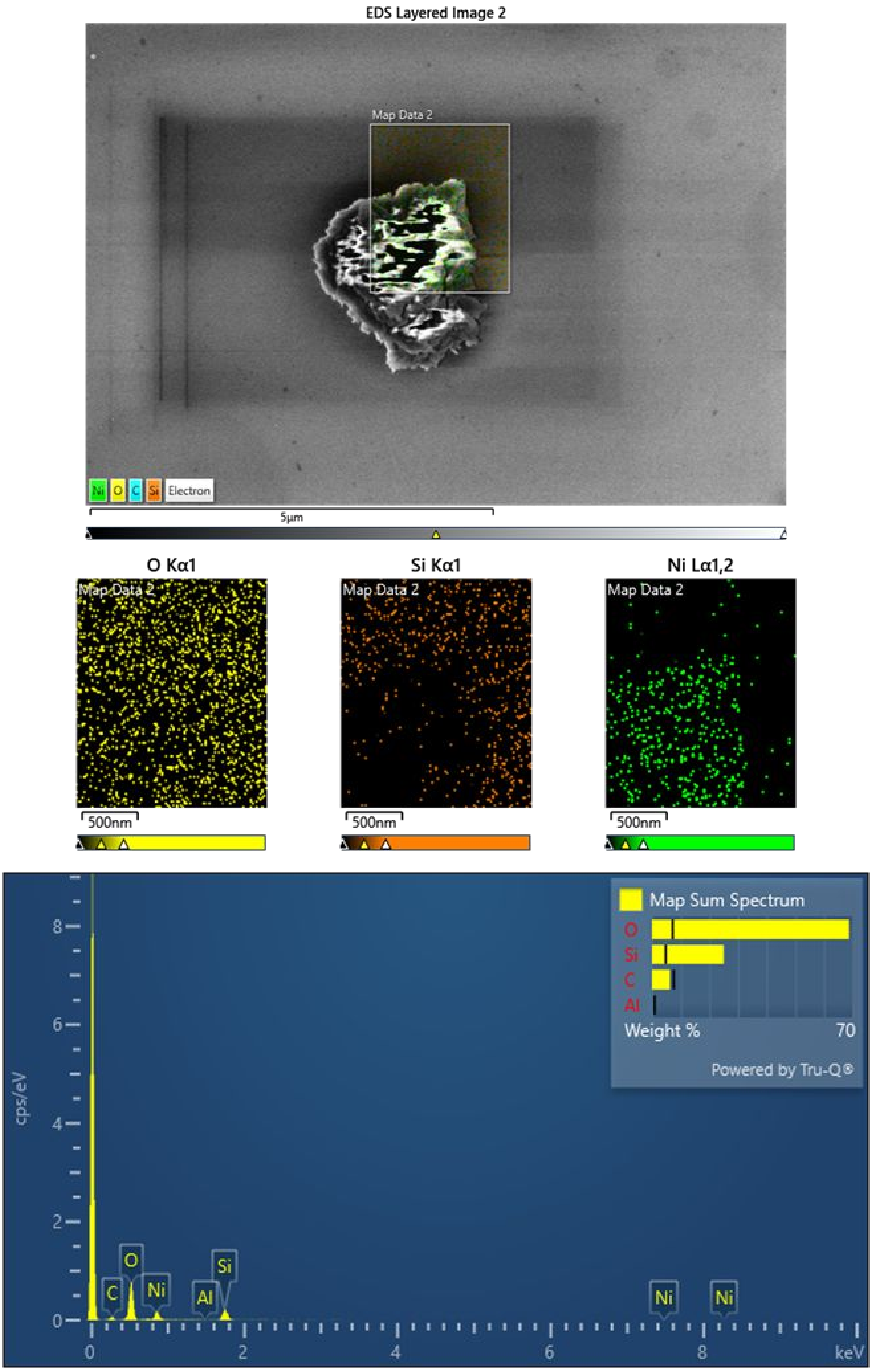

Figure S3. SEM image (top) and corresponding EDX mapping (bottom) of synthesized β-NiOOH.

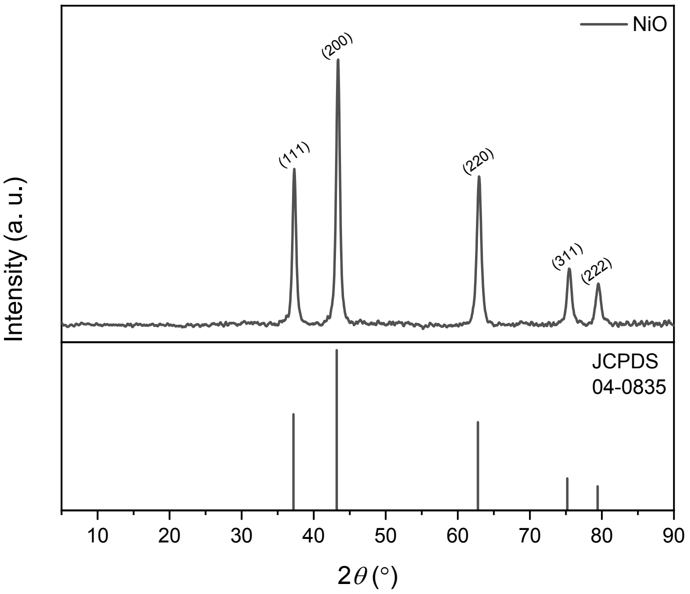

Figure S4. pXRD pattern of β-$Ni(OH)_2$ after TGA-DSC (5 K min–1, 25 to 400°C, 20 mL min–1 Ar) showing the selective formation of NiO.

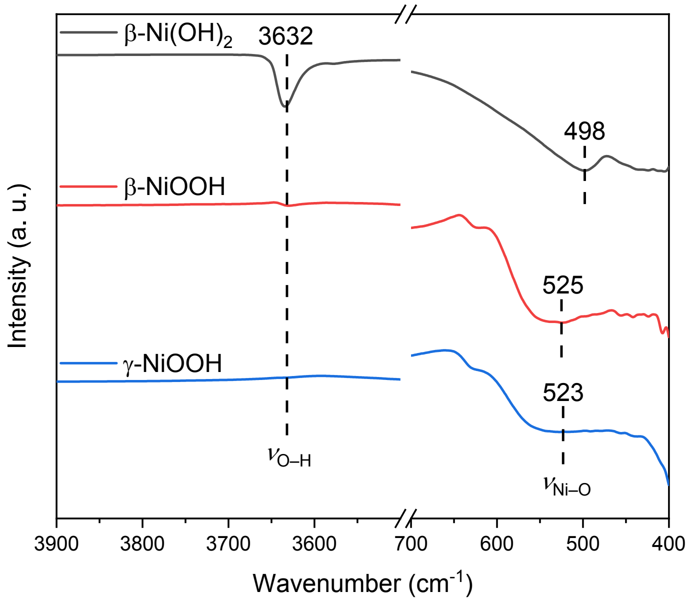

Figure S5. Fourier-transform infrared (FT-IR) spectra of β-Ni(OH)2, β-NiOOH and γ-NiOOH powder samples.

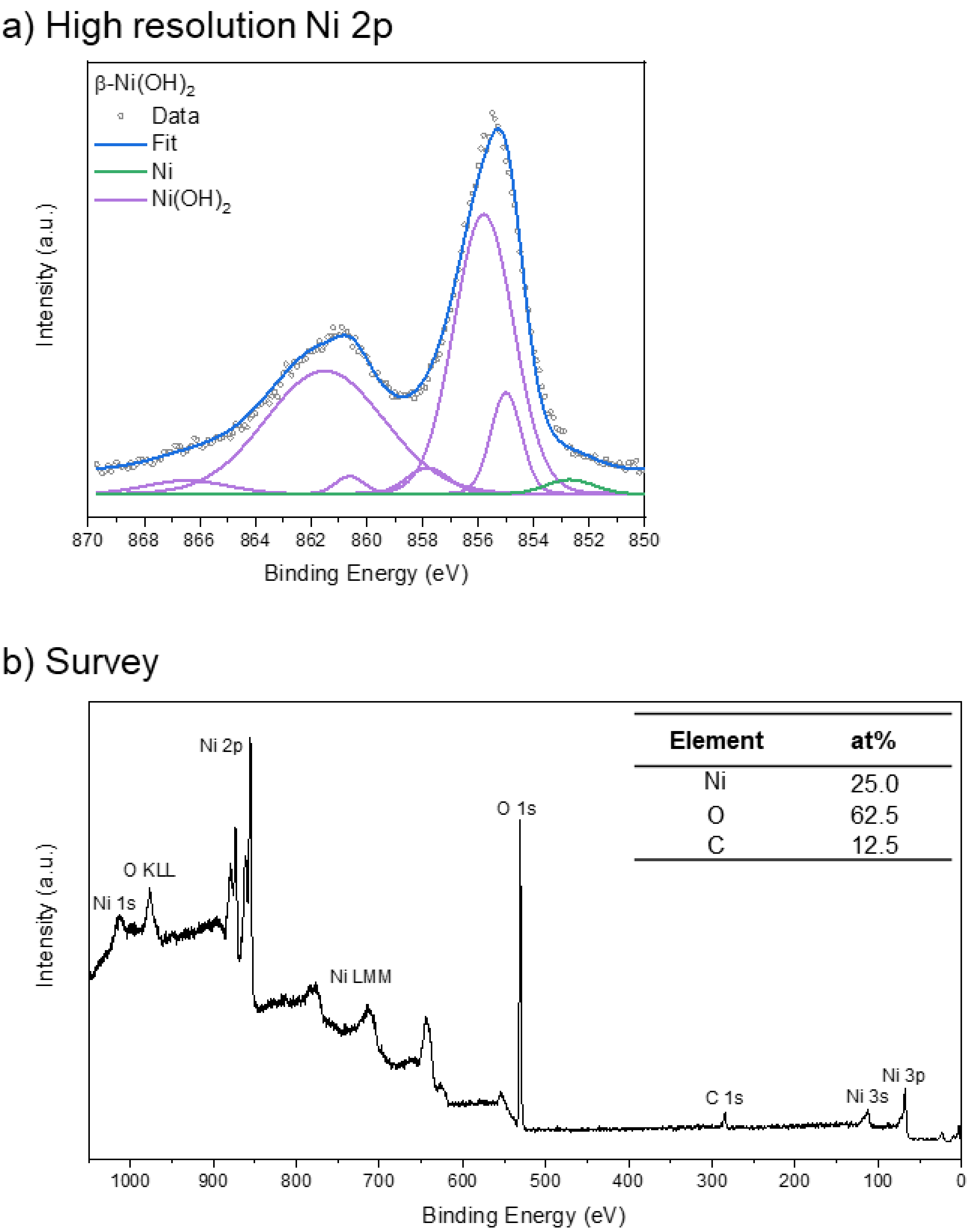

Figure S6. High-resolution spectra of the Ni $2p_{3/2}$ region (a) and survey XPS spectra (b) of commercial β-$Ni(OH)_2$. Inset shows the atomic ratio of the observed elements, not considering the difference in probing depth of the different core levels.

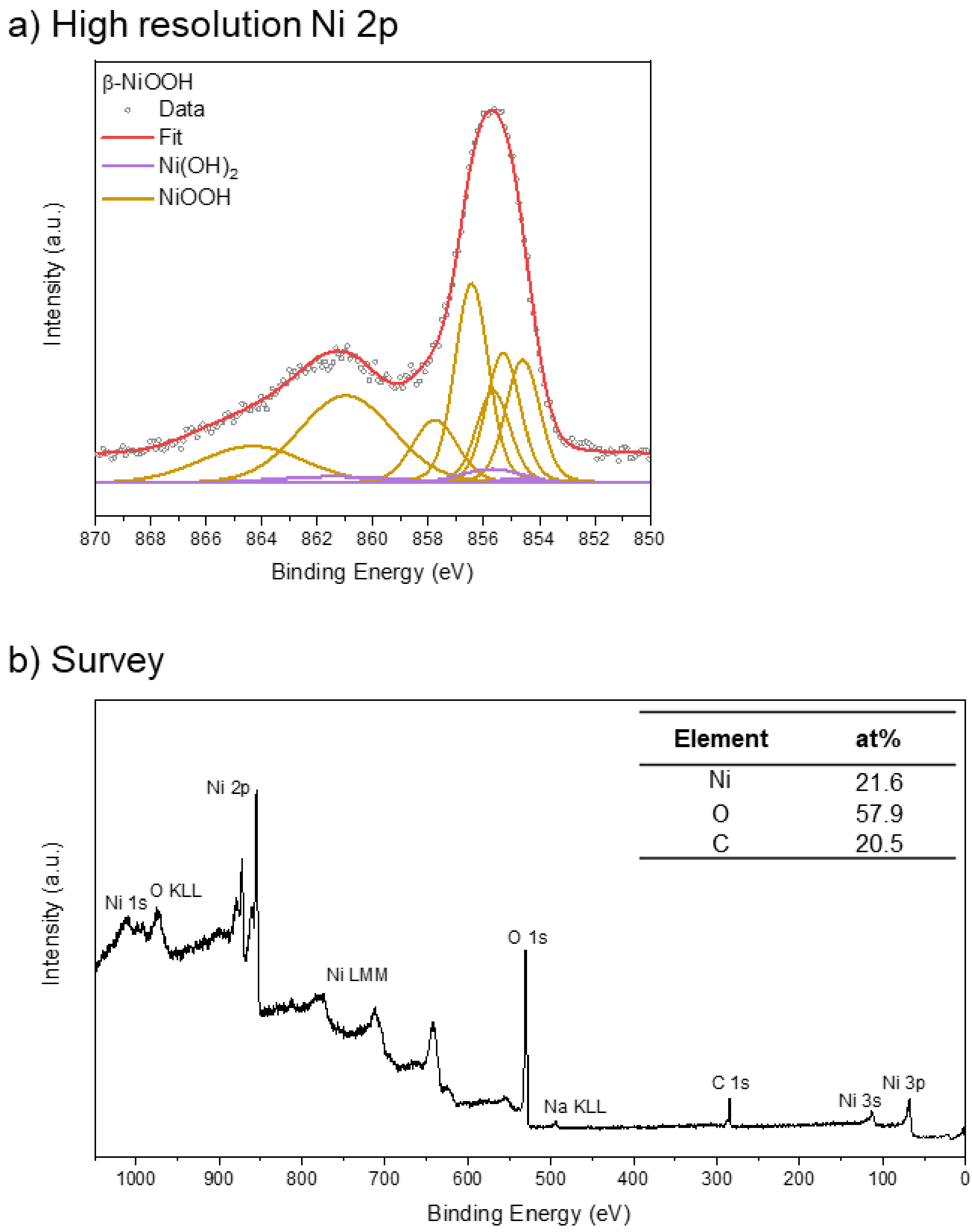

Figure S7. High-resolution spectra of the Ni $2p_{3/2}$ region (a) and survey XPS spectra (b) of synthesized β-NiOOH. Inset shows the atomic ratio of the observed elements, not considering the difference in probing depth of the different core levels.

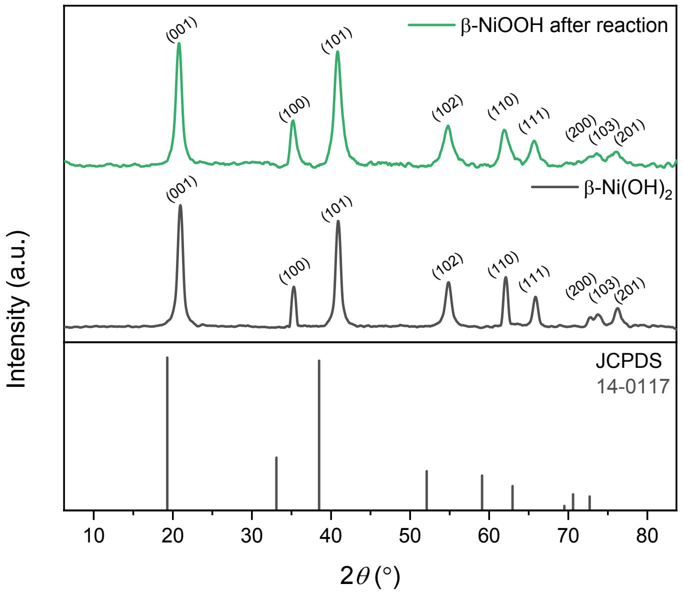

Figure S8. pXRD pattern of β-NiOOH (top, purple trace) after reaction with benzyl alcohol at room temperature in toluene for 1 hour, commercial β-$Ni(OH)_2$ (middle, black trace) and JCPDS line spectrum of β-$Ni(OH)_2$ (bottom, black trace).

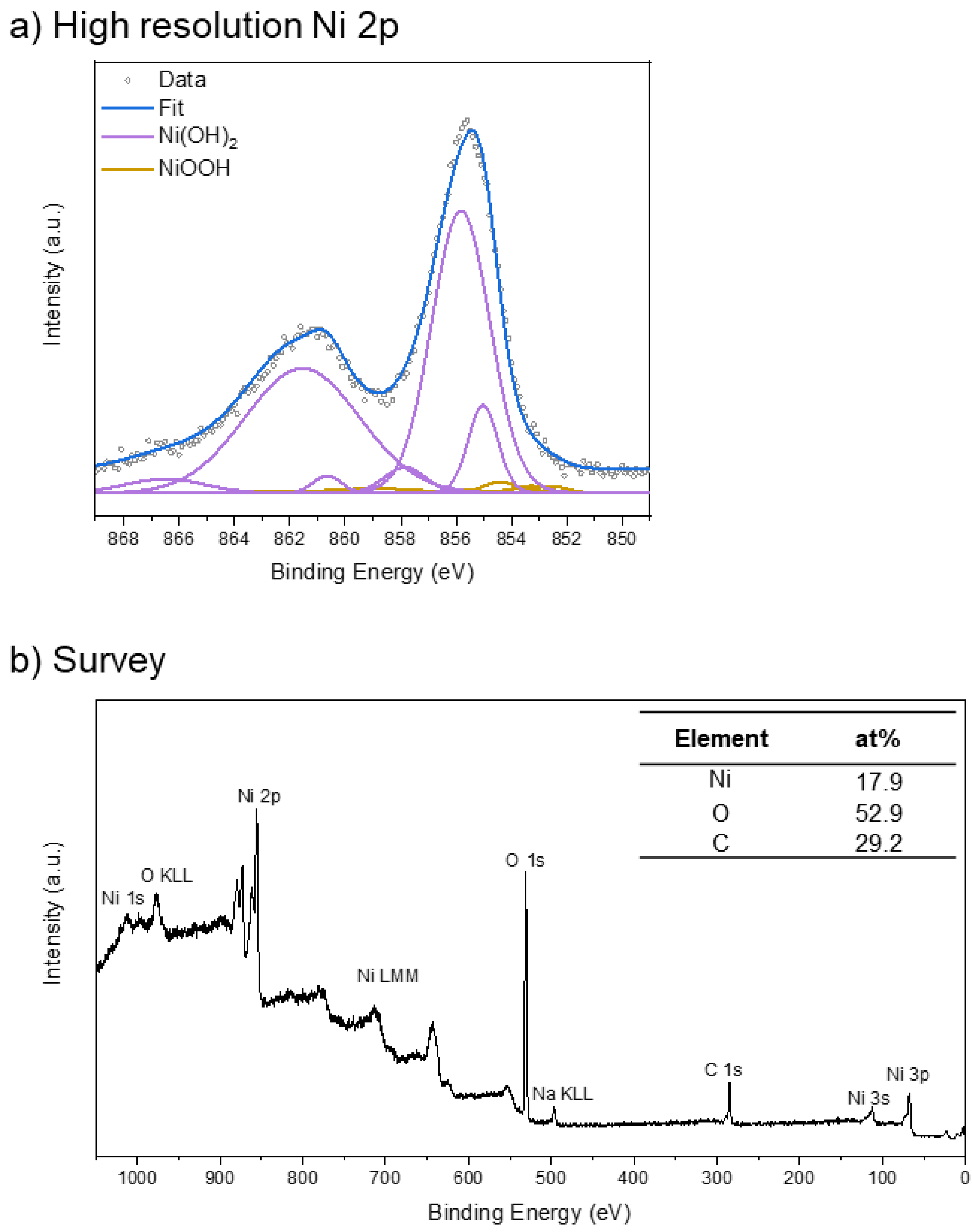

Figure S9. High-resolution spectra of the Ni $2p_{3/2}$ region (a) and survey XPS spectra (b) of β-NiOOH after reaction with benzyl alcohol showing the selective and quantitative conversion to β-$Ni(OH)_2$. Inset shows the atomic ratio of the observed elements, not considering the difference in probing depth of the different core levels.

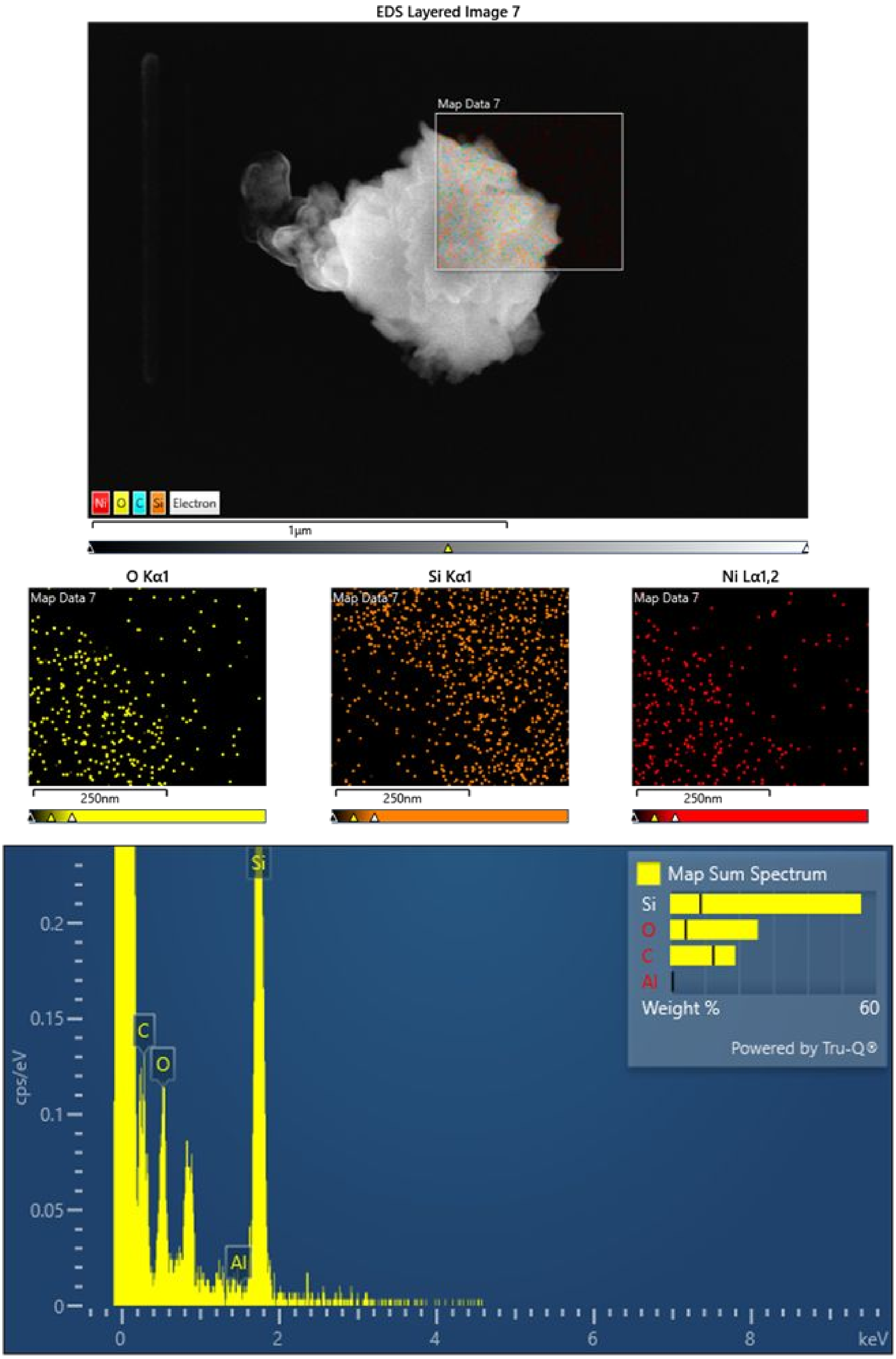

Figure S10. SEM image (top) and corresponding EDX mapping (bottom) of β-

NiOOH after reaction with benzyl alcohol at room temperature in toluene for 1 hour.

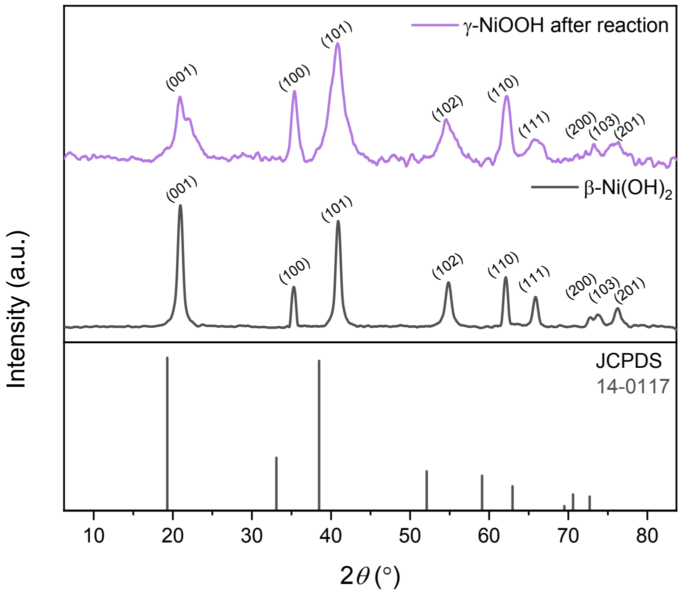

Figure S11. pXRD pattern of γ-NiOOH (top, purple trace) after reaction with benzyl alcohol at room temperature in toluene for 1 hour, commercial β-$Ni(OH)_2$ (middle, black trace) and JCPDS line spectrum of β-$Ni(OH)_2$ (bottom, black trace).

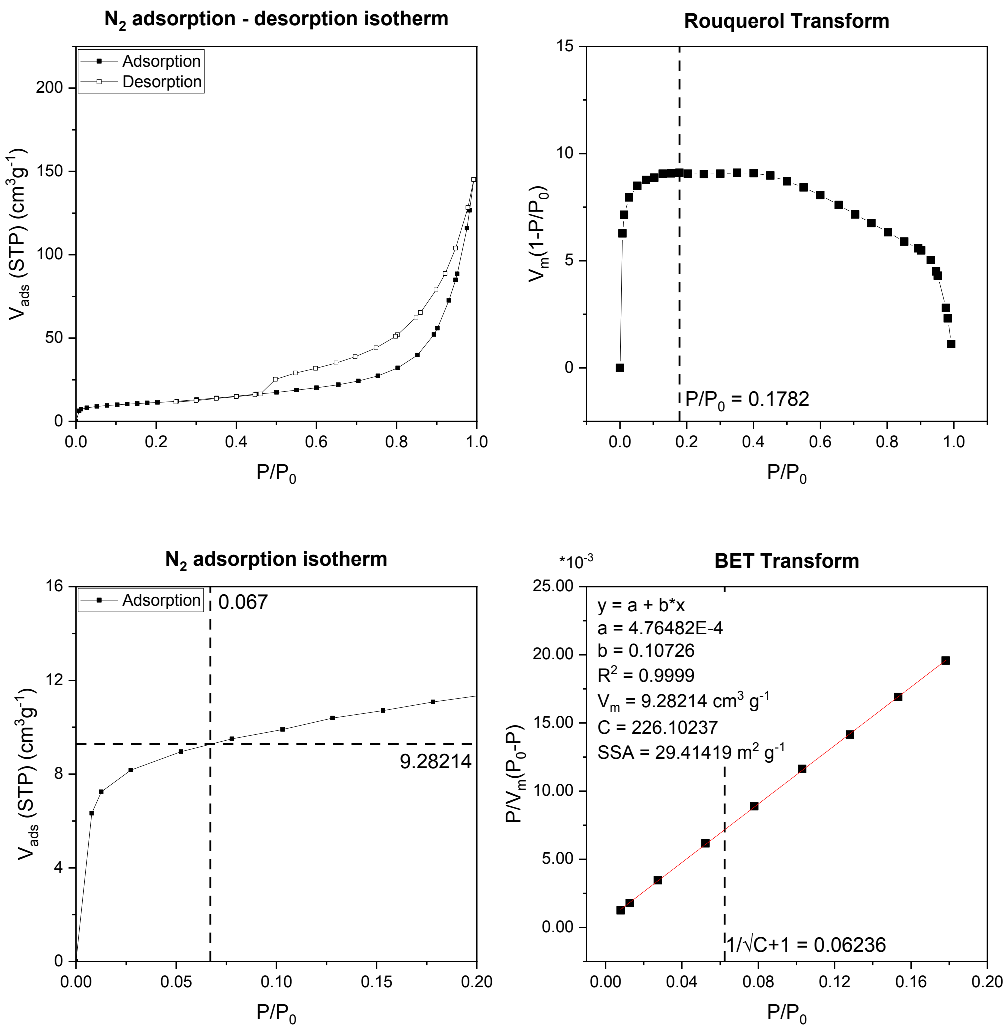

Figure S12. Porosity analysis of β-$Ni(OH)_2$. (a) N_2 adsorption and desorption isotherms at 77 K; (b) Rouquerol transform plot; (c) zoom-in of the N_2 adsorption isotherm at 77 K and (d) BET transform plot.

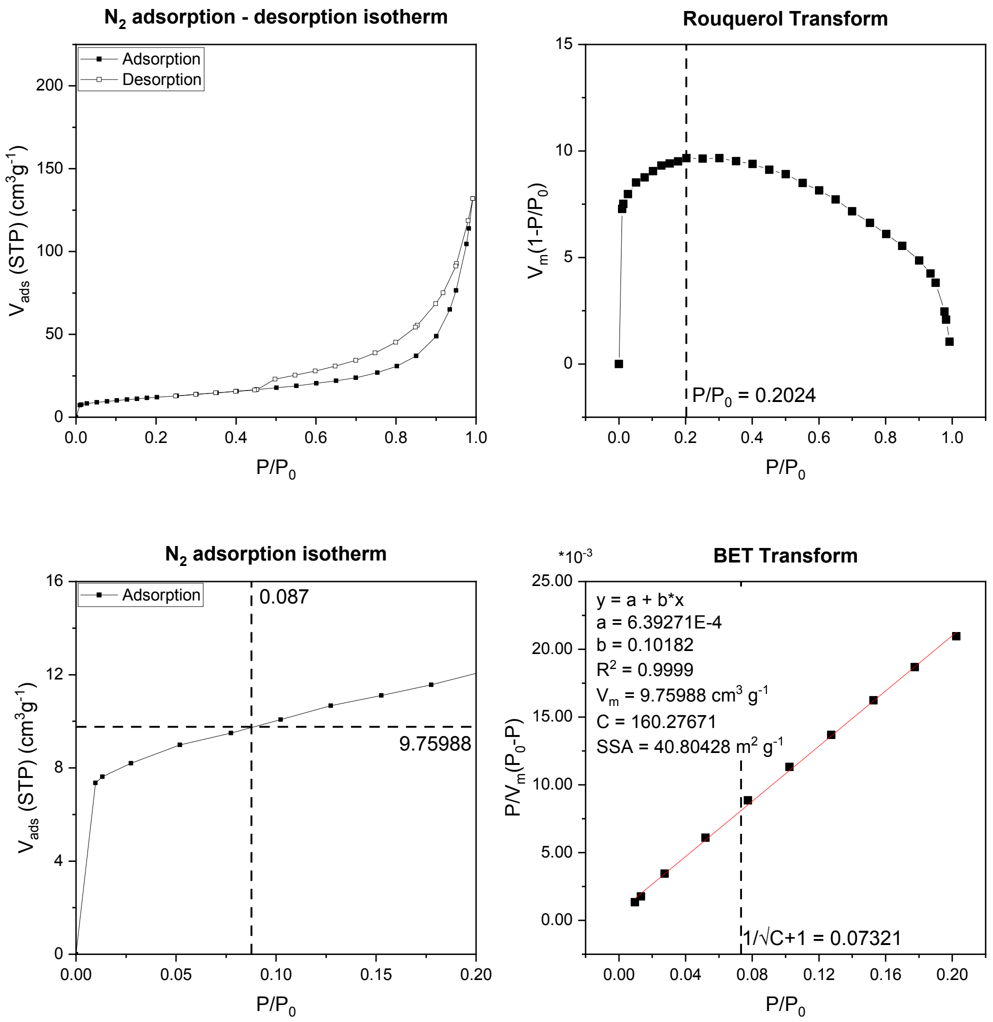

Figure S13. Porosity analysis of β-NiOOH. (a) N_2 adsorption and desorption isotherms at 77 K; (b) Rouquerol transform plot; (c) zoom-in of the N_2 adsorption isotherm at 77 K and (d) BET transform plot.

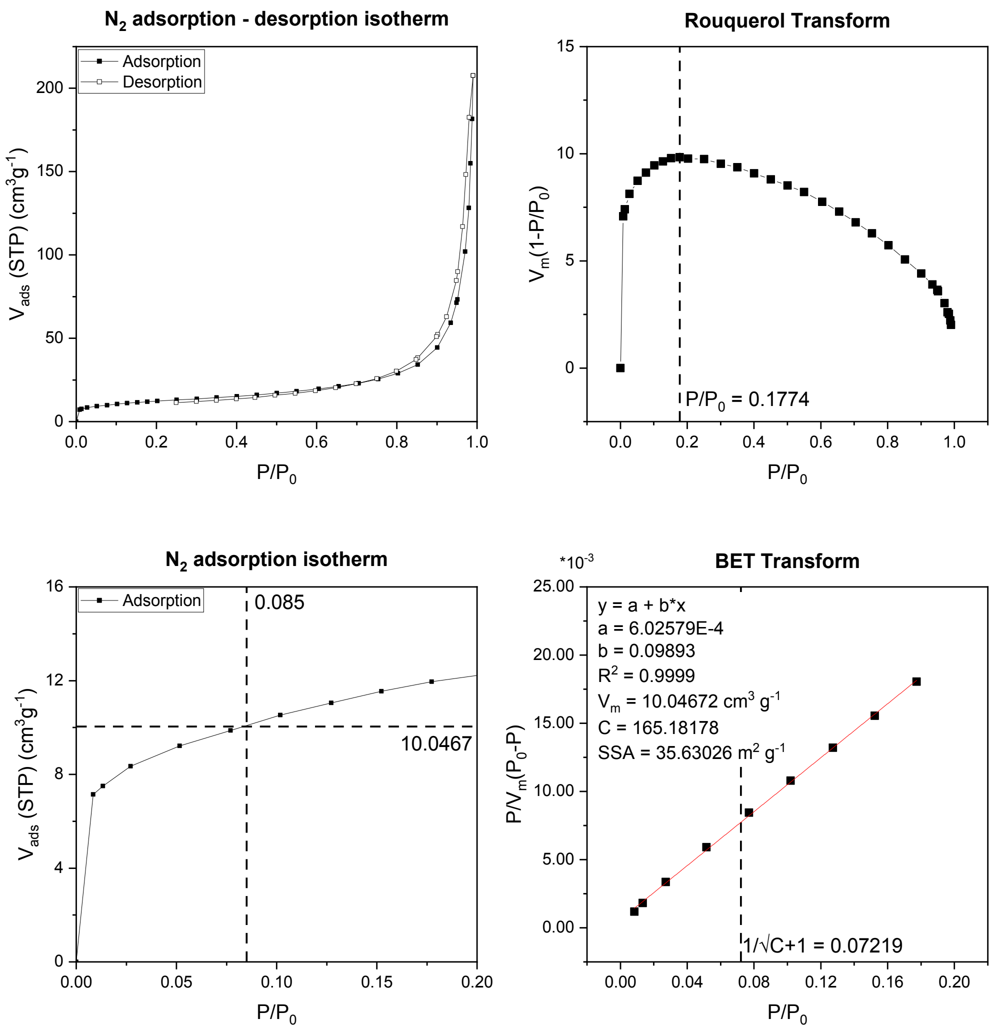

Figure S14. Porosity analysis of γ-NiOOH. (a) N_2 adsorption and desorption isotherms at 77 K; (b) Rouquerol transform plot; (c) zoom-in of the N_2 adsorption isotherm at 77 K and (d) BET transform plot.
